# Construction of Mussel-Inspired Dopamine–Zn^2+^ Coating on Titanium Oxide Nanotubes to Improve Hemocompatibility, Cytocompatibility, and Antibacterial Activity

**DOI:** 10.3389/fbioe.2022.884258

**Published:** 2022-03-31

**Authors:** Youdong Hu, Hualan Zhou, Tingting Liu, Minhui Yang, Qiuyang Zhang, Changjiang Pan, Jiafeng Lin

**Affiliations:** ^1^ The Second Affiliated Hospital and YuYing Children’s Hospital of Wenzhou Medical University, Wenzhou, China; ^2^ The Affiliated Huai’an Hospital of Xuzhou Medical University, Huai’an, China; ^3^ Faculty of Mechanical and Material Engineering, Jiangsu Provincial Engineering Research Center for Biomaterials and Advanced Medical Devices, Huaiyin Institute of Technology, Huai’an, China

**Keywords:** hemocompatibility, endothelialization, titanium oxide nanotubes, antibacterial activity, zinc ions

## Abstract

Zinc ions (Zn^2+^) are a highly potent bioactive factor with a broad spectrum of physiological functions. *In situ* continuous and controllable release of Zn^2+^ from the biomaterials can effectively improve the biocompatibility and antibacterial activity. In the present study, inspired by the adhesion and protein cross-linking in the mussel byssus, with the aim of improving the biocompatibility of titanium, a cost-effective one-step metal–catecholamine assembly strategy was developed to prepare a biomimetic dopamine–Zn^2+^ (DA-Zn^2+^) coating by immersing the titanium oxide nanotube (TNT) arrays on the titanium surface prepared by anodic oxidation into an aqueous solution containing dopamine (DA) and zinc ions (Zn^2+^). The DA-Zn^2+^ coatings with the different zinc contents exhibited excellent hydrophilicity. Due to the continuous release of zinc ions from the DA-Zn^2+^ coating, the coated titanium oxide nanotubes displayed excellent hemocompatibility characterized by platelet adhesion and activation and hemolysis assay. Moreover, the DA-Zn^2+^-coated samples exhibited an excellent ability to enhance endothelial cell (EC) adhesion and proliferation. In addition, the DA-Zn^2+^ coating can also enhance the antibacterial activity of the nanotubes. Therefore, long-term *in situ* Zn^2+^-releasing coating of the present study could serve as the bio-surfaces for long-term prevention of thrombosis, improvement of cytocompatibility to endothelial cells, and antibacterial activity. Due to the easy operation and strong binding ability of the polydopamine on various complicated shapes, the method of the present study can be further applied to other blood contact biomaterials or implantable medical devices to improve the biocompatibility.

## 1 Introduction

Cardiovascular disease aroused by vascular lesions and stenosis has become the leading threat to human health around the world, which puts forward great demand and higher requirements for the cardiovascular biomaterials and devices such as artificial heart valves and vascular stents. Due to their good corrosion resistance in physiological environments and the excellent mechanical properties, titanium-based biomaterials have been widely studied and applied in the cardiovascular implant, such as the artificial heart valve, vascular stent, and inferior vena cava filter; however, their surface biocompatibility needs to be further improved (Liu et al., 2004; Kaur and Singh, 2019; Sidhu et al., 2021). Generally speaking, when they come in contact with human blood and tissues, a series of side effects will occur on the titanium surface, such as the non-specific protein adsorption and denaturation, platelet adhesion and activation, endothelial dysfunction, or excessive proliferation of smooth muscle cells (Hedayati et al., 2019), which could prevent the formation of normal vascular endothelial layers on the surface and finally lead to thrombosis, delayed endothelial healing, and even implantation failure (Maitz et al., 2019). Therefore, it is of great significance to endow the titanium surface with the ability to regulate the responses of the physiological microenvironment through surface modification so as to inhibit thrombosis and induce endothelial cell growth.

For the titanium-based biomaterials used in the cardiovascular implants, although the current surface modification methods, such as surface chemical treatment (Aliofkhazraei et al., 2021), bioactive factor immobilization on the surface (Pan et al., 2014; Xu et al., 2021), and multi-functional biomimetic coating construction (Zhang et al., 2018; Liu et al., 2022), have obtained great progress in improving the biocompatibility and the ability to regulate the physiological microenvironment around the implant, the exogenous bioactive factors introduced by these methods are easy to inactivate in the *in vivo* physiological environment or in the process of the immobilization, and there is a risk of immune inflammation. Anodization is an easy and economical method to create micro-/nanostructures *in situ* on the titanium surface to enhance the biocompatibility without the introduction of the exogenous factors (Oliveira et al., 2017). The regular and robust titanium oxide nanotube arrays can be produced *in situ* on the titanium surface after the anodization (Gong et al., 2019), which can be further used to incorporate the bioactive factors to improve the biocompatibility so as to inhibit the occurrence of coagulation and promote the growth of endothelial cells.

**FIGURE 1:**
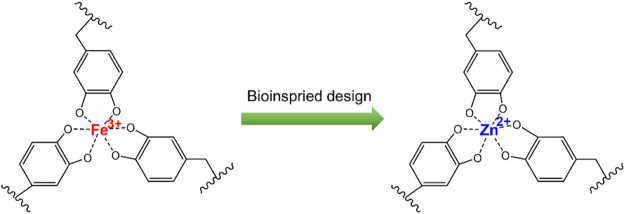
Schematic diagram of the formation of the dopamine–Zn^2+^ coating.

Zinc is a necessary trace element in the human body, and it participates in regulating many human physiological responses. Zinc exists in more than 3,000 human proteins and can effectively regulate cell growth behaviors and functional expression (Krężel and Maret, 2016). It has been reported that zinc deficiency *in vivo* is closely related to atherosclerosis (Beattie and Kwun, 2004), and zinc can also protect the integrity of vascular endothelial cells (Choi et al., 2018). Therefore, the introduction of zinc on the titanium surface can promote the formation of the vascular endothelium. In addition, zinc ions also exhibit good antibacterial activity due to their ability to induce the bacterial decomposition by changing the charge balance (Li et al., 2019; Chen et al., 2021). Consequently, loading zinc ions (Zn^2+^) into the anodized titanium oxide nanotubes can effectively inhibit the bacterial infection and promote the growth of endothelial cells. In this study, inspired by the adhesion and protein cross-linking chemistry of [Fe(DA)_3_] complexes found in the mussel byssus coatings, a dopamine–Zn^2+^ coordination complex was one-step-produced on the anodized titanium oxide nanotube surface, according to the characteristics that dopamine can chelate metal ions to form a tight polydopamine coating on the substrate surface (Figure 1). The results showed that the surface coatings with different Zn^2+^ amounts can be easily constructed by this metal catecholamine assembly strategy, which can significantly improve the hemocompatibility, endothelial cell growth, and antibacterial activity of the titanium surface. Because dopamine can be easily bound to the surface of the solid materials with various complex shapes, the method in this study can be further applied to other blood-contacting biomaterials or implantable medical devices to improve the biocompatibility.

## 2 Materials and Methods

### 2.1 Construction of Dopamine–Zn^2+^ Coatings on Titanium Oxide Nanotubes

First, the titanium oxide nanotube (TNT) arrays were prepared *in situ* on the pure titanium surface (TA2) by anodic oxidation. The titanium plate was used as the anode, and the graphite rod was used as the cathode. The anodic oxidation was carried out in an ethylene glycol solution containing 5 wt% NH_4_F and 2 vol% H_2_O. The oxidation voltage was 45 V, and the current value was 3 A. After 1 h, the oxidized sample was ultrasonically cleaned by ethylene glycol and ethanol for 5 min each. In order to enhance the binding force between the substrate and the oxidized layer and change the crystal structure of the titanium oxide nanotubes, the as-prepared sample was dried and then treated for 2.5 h at 450°C under atmospheric conditions to obtain the annealed TNT (TNTA).

In order to construct the dopamine–Zn^2+^ (DA-Zn^2+^) coatings with different Zn^2+^ concentrations on the TNTA surface, 0.05, 0.1, and 0.15 g ZnSO_4_.7H_2_O was added into a 50 ml dopamine solution (2 mg/ml in Tris–HCl buffer, pH 8.5), respectively, and then the TNTA samples were immersed into the mixed solution for 12 h to obtain the DA-Zn^2+^ coatings on the titanium oxide nanotubes; the final samples were labeled as TNTA-Zn1, TNTA-Zn2, and TNTA-Zn3, respectively.

### 2.2 Surface Characterization

The surface morphologies of the titanium oxide nanotubes were observed by scanning electron microscopy (SEM, FEI Quata 250), and the surface element concentrations were detected by energy dispersive X-ray spectrometry (EDS, IMAX-Max 20, Britain). In order to further observe the cross-sectional morphology, the sample was cut and observed by SEM. The surface chemical structures of the different samples were characterized by attenuated total reflection Fourier transform infrared spectroscopy (ATR–FTIR, TENSOR 27, Bruker, Germany). The water contact angles were measured on a DSA25 contact angle measuring instrument (Krüss GmbH, Germany) to characterize the surface wettability. Three parallel samples were measured and the values were averaged.

### 2.3 Zinc Ions Release Profiles of DA-Zn^2+^ Coatings

The dopamine–Zn^2+^-coated samples were immersed into a 10 ml phosphate-buffered solution (PBS) at 37°C under stirring. At predetermined intervals, 0.5 ml PBS was taken and supplemented with 0.5 ml fresh PBS. The Zn^2+^ concentration was determined by an inductively coupled plasma emission spectrometer (Optima 7000 DV). The ion release profiles of the different samples were plotted according to the standard curve. For each kind of sample, three parallel samples were used for measurements and the values were averaged.

### 2.4 Protein Adsorption

The adsorption amounts of bovine serum albumin (BSA) and fibrinogen (FIB) on the different surfaces were measured by the BCA method. The BCA kit, BSA, and FIB were purchased from Shenzhen Ziker Biological Technologies Co., Ltd. Briefly, the sample with a dimension of 10 × 10 mm was first equilibrated for 2 h with PBS to reduce the measurement errors and then dipped into 1 mg/ml BSA and FIB solutions, respectively. After being immersed for 3 h at 37°C, the sample was rinsed three times with PBS, followed by immersing it into a 2 ml 1 wt% sodium dodecyl sulfate solution (SDS) to ultrasonically desorb for 30 min. Then 100 μl eluent and 100 μl BCA working solution were thoroughly mixed for 20 min at 37°C, and the 150 μl mixture was transferred to a 96-well plate to measure the absorbance at 562 nm. The amount of the adsorbed protein was calculated according to the standard curves.

### 2.5 Anticoagulation of the DA-Zn^2+^-Coated Samples

#### 2.5.1 Platelet Adhesion and Activation

The fresh whole blood containing citrate sodium as the anticoagulant from a healthy volunteer was centrifuged for 10 min at 1,500 rev to obtain the platelet-rich plasma (PRP). 200 μl PRP was dropped on each sample to cover the whole surface and then incubated for 2 h at 37°C. After that, the samples were rinsed twice with the physiological saline to remove the non-adherent platelets, and the attached platelets were fixed for 3 h at 4°C by a 2.5% glutaraldehyde solution. The samples were successively dehydrated with 50, 70, 90, and 100% ethanol solutions for 15 min each. After drying under atmospheric conditions, the attached platelets on the surface were observed by SEM (FEI Quanta250).

The platelet activation was characterized by detecting the granule membrane glycoprotein of platelet (GMP140) using an enzyme linked immunosorbent assay kit (ELISA, Shanghai Enzyme-Linked Biotechnology Co., Ltd.). First, 50 μl PRP was dropped on the sample surface and incubated for 2 h at 37°C. Subsequently, 10 μl cultured PRP and 40 μl diluent were added into a 96-well plate, followed by adding 100 μl ELISA reagent and culturing for 1 h at 37°C. After rinsing five times, 50 μl chromogenic agent A and 50 μl chromogenic agent B were successively added to react for 15 min in the dark. Finally, a 50 μl stopping solution was added to terminate the reaction. The absorbance at 450 nm was determined, and the GMP140 was calculated according to the standard curve.

#### 2.5.2 Hemolysis Rate

The fresh whole blood from a healthy volunteer was centrifuged for 10 min at 1,500 rev to isolate the red blood cells. The red blood cells were diluted into 2% suspension using 0.9% physiological saline. A 2 ml red blood cell suspension was incubated for 3 h with a different sample at 37°C, and then a 1 ml cultured suspension was taken out to centrifuge for 5 min at 3,000 rev. A 200 µl supernatant was transferred into a 96-well plate to measure the absorbance at 450 nm. The 2% red blood cell suspension in the physiological saline and the distilled water were used as the positive control and the negative control, respectively. The hemolysis rate was calculated according to the following equation:

Hemolysis rate (%) = D0 - D2/D1- D2×100%,

where D0, D1, and D2 represent the absorbance of the sample, positive control, and negative control, respectively.

### 2.6 Endothelial Cell Adhesion and Proliferation

The sterilized samples were first placed into a 24-well plate, and then 0.5 ml endothelial cell suspension (ECV304, 5 × 10^4^ cells/ml) and 1.5 ml DMEM culture medium were added. After culturing for 1 day and 3 days at 37°C and 5% CO_2_, respectively, the samples were washed three times with PBS to remove the non-attached cells. The attached cells were fixed for 3 h at 4°C by a 2.5% glutaraldehyde solution. Subsequently, the cells were stained with 200 μl rhodamine (10 μg/ml in PBS) for 30 min and 200 μl DAPI (500 ng/ml in PBS) for 8 min, respectively. The fluorescence images were taken by a Zeiss Inverted A2 microscopy to observe the behaviors of cell adhesion.

For cell proliferation, the endothelial cells were cultured as described previously. After 1 day and 3 days, respectively, the samples were rinsed with PBS, and then a 0.5 ml CCK-8 solution (CCK8:culture medium = 9:1) was added for incubating for 3.5 h at 37°C. Subsequently, the 200 μl supernatant was transferred into a 96-well plate to measure the absorbance at 450 nm by a micro-plate reader (Bio-Tek, Eons). Three parallel samples were used for CCK-8 assay in order to reduce the errors.

### 2.7 Antibacterial Activity

*Escherichia coli* (*ATCC 25922,* Shanghai YaJi Biological Co. Ltd.) was used as the model bacterium to characterize the antibacterial activity of the samples. The samples were first placed into a 12-well plate, and then a 500 μl bacterial solution (5 × 10^4^ CFU/ml) was added to incubate for 30 min at 37°C. After that, 1,500 μl sterilized deionized water was added and cultured for 24 h at 37°C. Finally, 50 μl bacterial liquid from the plate was covered on the solid medium surface to culture overnight at 37°C. The bacteria were observed by taking pictures using a HUAWEI Nova 6 smartphone.

## 3 Results and Discussion

### 3.1 Surface Characterization

**FIGURE 2:**
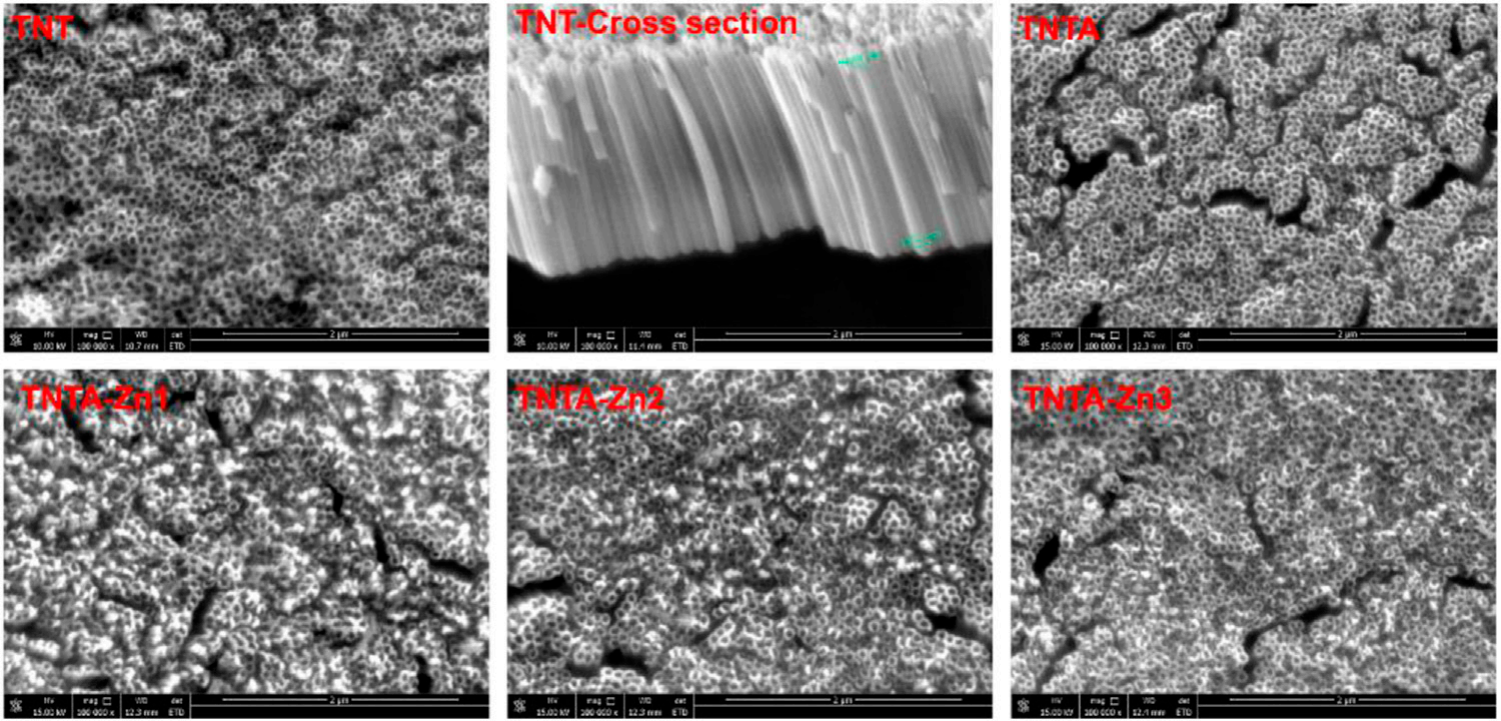
Surface morphologies of the different samples characterized by SEM (scale bar is 2 μm).

Titanium and its alloys have been extensively explored for the blood-contacting biomaterials and implants. In the present study, in order to improve the biocompatibility, the pure titanium plate was first treated by the anodic oxidization to produce the regular titanium oxide nanotubes on the surface, and the DA-Zn^2+^ coating on the titanium oxide nanotubes was further constructed by one-step dip-coating of the samples into an alkaline aqueous solution containing Zn^2+^ and dopamine. To obtain the coatings with adjustable and controllable release of zinc ions, DA-Zn^2+^ coatings were produced on the titanium oxide nanotubes by immersing the samples into the dopamine solution with various ZnSO_4_.7H_2_O feed amounts. The surface morphologies of the anodized nanotubes and the DA-Zn^2+^-coated nanotubes were first observed by SEM. As shown in Figure 2, it can be seen that the regular nanotube arrays with about 100 nm diameter and 2 μm length were successfully constructed on the titanium surface. After annealing treatment at 450°C, the surface morphologies of the nanotubes did not obviously change. According to our previous study (Gong et al., 2019), the amorphous titanium oxide nanotube after the anodization could change into the anatase crystal structure when treated at 450°C, which could enhance the surface hydrophilicity and biocompatibility because of more oxygen introduction and the removal of the hydrophobic fluoride element. The diameter of the nanotubes became smaller and some nanotubes were even blocked after the construction of dopamine–Zn^2+^ coating, and the surface also became rougher, indicating that the DA-Zn^2+^ coatings were successfully prepared on the surfaces. The surface element concentrations were further examined by EDS, and the results are shown in Table 1. More fluoride elements can be detected on the TNT surface because in the process of anodic oxidation, fluoride ions (F^−^) in the electrolyte can form (NH_4_)_2_TiF_6_ on the surface through etching the titanium oxide layer (Regonini et al., 2013). On the other hand, the ratio of Ti and O elements on all samples was close to 3:2, demonstrating that the oxide film on the surface was mainly TiO_2_. In the process of the annealing treatment, NH_4_F can be generated from (NH_4_)_2_TiF_6_ on the TNTA surface (Liu et al., 2009), resulting in the removal of the fluoride element. Therefore, only 0.5% fluoride can be found on the TNTA surface; concurrently, more oxygen can be introduced on the surface. Due to the occurrence of DA-Zn^2+^ coatings on the surface, C, Zn, and N can be detected at the same time, suggesting that the DA-Zn^2+^ coating was successfully created on the surface. With the increase in Zn^2+^ feed in the dopamine solution, the zinc concentration in the coatings increased; however, the N concentration did not change significantly. In the present study, the dopamine concentration was fixed, and a different amount of Zn^2+^ was added to form the DA-Zn^2+^ coating with different Zn^2+^ contents. Therefore, the zinc concentration increased while the N element did not change significantly with the increase in zinc ions.

**TABLE 1:** Surface element concentration (wt%) of the different samples characterized by EDS.

	Ti	O	F	C	Zn	N
TNT	64.4	25.3	10.3	—	—	—
TNTA	58.9	40.6	0.5	—	—	—
TNTA-Dopa-Zn1	54.6	37.2	0.2	4.8	1.8	1.4
TNTA-Dopa-Zn2	55.4	36.5	0.2	4.2	2.4	1.3
TNTA-Dopa-Zn3	52.4	37.4	0.1	5.5	3.1	1.5

**FIGURE 3:**
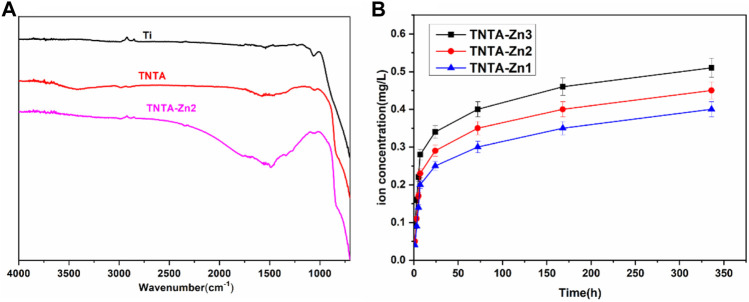
FTIR spectra **(A)** of the different samples and the release profiles of zinc ions **(B)**.

In order to further investigate the changes of the surface chemical groups, ATR–FTIR was carried out and the results are shown in Figure 3A. It can be clearly seen that no infrared adsorption can be detected on the pristine titanium surface because there were no chemical groups on it. The anodization and the following annealing treatment can introduce more oxygen in the nanotubes (Table 1), which could produce many hydroxyls on the surface; consequently, a board adsorption peak can be found at 3,200–3,400 cm^−1^ on the TNTA surface, which can be attributed to the adsorption of the hydroxyls. After the construction of the DA-Zn^2+^ coating, the appearance of C=C at 1,580 cm^−1^ and C-H at 1,480 cm^−1^ clearly indicated the existence of benzene ring, which belongs to the polydopamine coating. The C=O adsorption peak at 1,730 cm^−1^ can be found on the TNTA-Zn2 surface, suggesting that a relative complicated structure was formed on the surface. At the same time, no amine groups can be detected on the surface, suggesting that the amine of dopamine could participate in chelating with zinc ions and the polydopamine was formed on the surface.

The release profiles of zinc ions from the different DA-Zn^2+^ coatings were characterized by immersing the samples into PBS to collect Zn ions. As shown in Figure 3B, the total amount of zinc ions released from the different samples after 14 days were 0.41 mg/L for TNTA-Zn1, 0.45 mg/L for TNTA-Zn2, and 0.51 mg/L for TNTA-Zn3, respectively. With the increase in the zinc feed concentration in a dopamine solution during DA-Zn^2+^ coating deposition, the zinc ion concentration released from the titanium oxide nanotubes increased slightly. According to the results of element analysis (Table 1), it can be concluded that a higher zinc amount in the coating can contribute to a higher zinc ion release. In addition, it can be seen from Figure 3B that Zn^2+^ release had a burst release behavior in about 7 h, and the zinc ion release amount was 0.2–0.28 mg/L for the different samples, which represented about 50% of the total amount of 14 days. It was possible that some zinc ions were not chelated with polydopamine coating, which resulted in the burst release. The release of zinc ions entered a slow stage after 7 h. During this period, the Zn^2+^ release rate from the nanotubes with the larger Zn^2+^ loading was faster than that of smaller zinc loading. In the process of coating preparation, the chemical valence of zinc ions did not change and zinc was divalent in the coating; therefore, the decomposition and degradation diffusion from the coating may be the major mechanism of zinc release. After the burst release, a stable release profile can be observed because the zinc ions from the nanotubes are mainly released by the degradation diffusion. By the seventh day, the content of zinc ion released nearly 85–91% of the 14 day release, and the Zn^2+^ release rate was gradually stabilized for all samples.

### 3.2 Surface Wettability and Protein Adsorption

**FIGURE 4:**
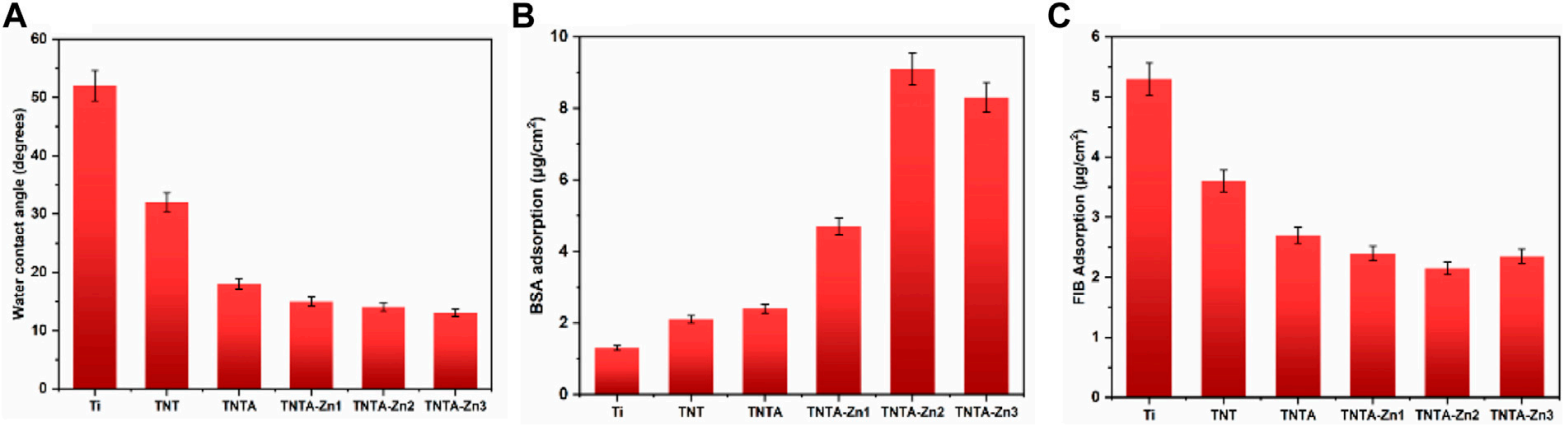
Water contact angles **(A)**, BSA adsorption **(B)**, and FIB adsorption **(C)** of the different samples. Three parallel samples were measured, and the values were averaged and are expressed as mean ± SD (standard derivation).

Surface wettability represents an important factor affecting the biocompatibility of biomaterials (Rahimipour et al., 2020). Generally speaking, the hydrophilic surface can enhance cell adhesion and proliferation and improve the blood compatibility when compared to the hydrophobic surface because most of the human tissues have plenty of water and are in the hydrophilic environment (Liu et al., 2007). In order to explore the relationship between the surface wettability and the biocompatibility of the titanium oxide nanotubes before and after the introduction of the DA-Zn^2+^ coating, the water contact angles of the different samples were measured to characterize the surface wettability. As shown in Figure 4A, it can be seen that the water contact angle was reduced from 52.4° of the blank titanium to 32.5° of TNT, indicating that the titanium dioxide nanotubes have good hydrophilicity. It has been reported that the annealing treatment of the anodized titanium oxide nanotubes can remove the organic pollutants and thus enhance the hydrophilicity of the TNT surface (Chen et al., 2014; Lorenzetti et al., 2015). At the same time, after the annealing treatment, the hydrophobic fluorine elements can be almost removed (Table 1), along with the introduction of the hydrophilic hydroxyls (Figure 3A) and more oxygen elements in the nanotubes, which also contributed to the improvement of the hydrophilicity. In addition, it is well known that the amorphous TNT after the anodization can be changed into the anatase crystal structure after being treated at 450°C, which has better hydrophilicity than the amorphous TiO_2_ nanotubes (Varghese et al., 2003). Consequently, the hydrophilicity of TNTA was significantly improved, and the water contact angle was further reduced to 18.7°. After the preparation of the DA-Zn^2+^ coating, the water contact angle decreased slightly as compared to that of TNTA. On the one hand, the surface polydopamine coating can form the hydrogen bonds with the water molecules to improve the hydrophilicity (Qiu et al., 2018). On the other hand, after the DA-Zn^2+^ coating was prepared on the surface, the surface morphology became rougher and the diameter of the nanotubes became smaller, which can reduce the action of the capillary effect of the nanotubes and thus prevent more water molecules penetrating into the nanotubes, leading to reduced hydrophilicity. Consequently, the water contact angles of the DA-Zn^2+^-coated nanotubes did not change significantly as compared to those of TNTA.

Protein adsorption plays an important and sometimes determined role in the biological behaviors of the implantable biomaterials because it represents the first event when biomaterials are implanted in the human body (Brash et al., 2019). Fibrinogen and albumin are two major proteins in the human blood, which have significant influences on the blood compatibility and cell behaviors (Nonckreman et al., 2010; Chen et al., 2016). In general, for blood-contacting biomaterials and implants, albumin adsorption can inhibit the platelet adhesion and activation on the biomaterials surface and thus inhibit the occurrence of the thrombus (Xu et al., 2014). However, the fibrinogen absorption and denaturation on the surface could promote the platelet adhesion and activation, and even lead to the formation of thrombus (Wang et al., 2021). The results of albumin adsorption on the different samples are shown in Figure 4B. It can be seen that due to the specific surface morphology, the nanostructure of the nanotubes can enhance the albumin adsorption to some extent as compared to the pristine titanium. It was considered that the increased specific surface area and the negative charges on the surface after the anodization can contribute to the attachment of the positive charged albumin. The annealing treatment further enhanced albumin adsorption due to the crystal structure changes and better hydrophilicity of TNTA; however, its effect was not obvious. In this study, zinc ions were loaded into the nanotubes by the dopamine chelation reaction; the polydopamine coating can significantly enhance the protein adsorption. Moreover, the DA-Zn^2+^-coated titanium oxide surface became rougher than the TNTA surface, which can also contribute to the enhancement of albumin adsorption. Therefore, the DA-Zn^2+^ can significantly enhance the albumin adsorption. With the increase in zinc ions, the amount of albumin adsorption was increased; however, there was no difference between TNTA-Zn2 and TNTA-Zn3, indicating that the BSA adsorption on the surface may be saturated.

The fibrinogen adsorption amounts of the different samples are displayed in Figure 4B. It can be seen that more fibrinogen can be adsorbed on the pristine titanium surface because fibrinogen tends to be adsorbed on the hydrophobic surface (Wu et al., 2021). The fibrinogen concentration adsorbed on the TNT surface decreased rapidly because the hydrophilicity was significantly improved after the anodization. The fibrinogen adsorption further decreased on the TNTA surface due to its better hydrophilicity and the increased thickness of the oxide layer after the annealing treatment (Sharma and Paul, 1992). Our previous results showed that the increase in the titanium dioxide nanotube diameter can enhance fibrinogen adsorption (Gong et al., 2019). It can be seen from Figure 2 that the DA-Zn^2+^ coating can significantly reduce the nanotube diameter, which can contribute to prevent fibrinogen adsorption to some degree. On the other hand, the increased hydrophilic surface of the DA-Zn^2+^ coating on the nanotubes can also inhibit fibrinogen adsorption because the water layer could prevent fibrinogen diffusing to the surface. It was noteworthy that there was no obvious difference for albumin and fibrinogen on the DA-Zn^2+^ coatings with different zinc concentrations. This may be because the three coatings had similar hydrophilicity, morphology, and nanotube diameter. In addition, the DA-Zn^2+^ coating can inhibit the fibrinogen adsorption while promoting the adsorption of albumin, showing that it had the character of the selective albumin adsorption, which is conducive to improve the blood compatibility.

### 3.3 Effects of DA-Zn^2+^ Coating on Blood Compatibility

**FIGURE 5:**
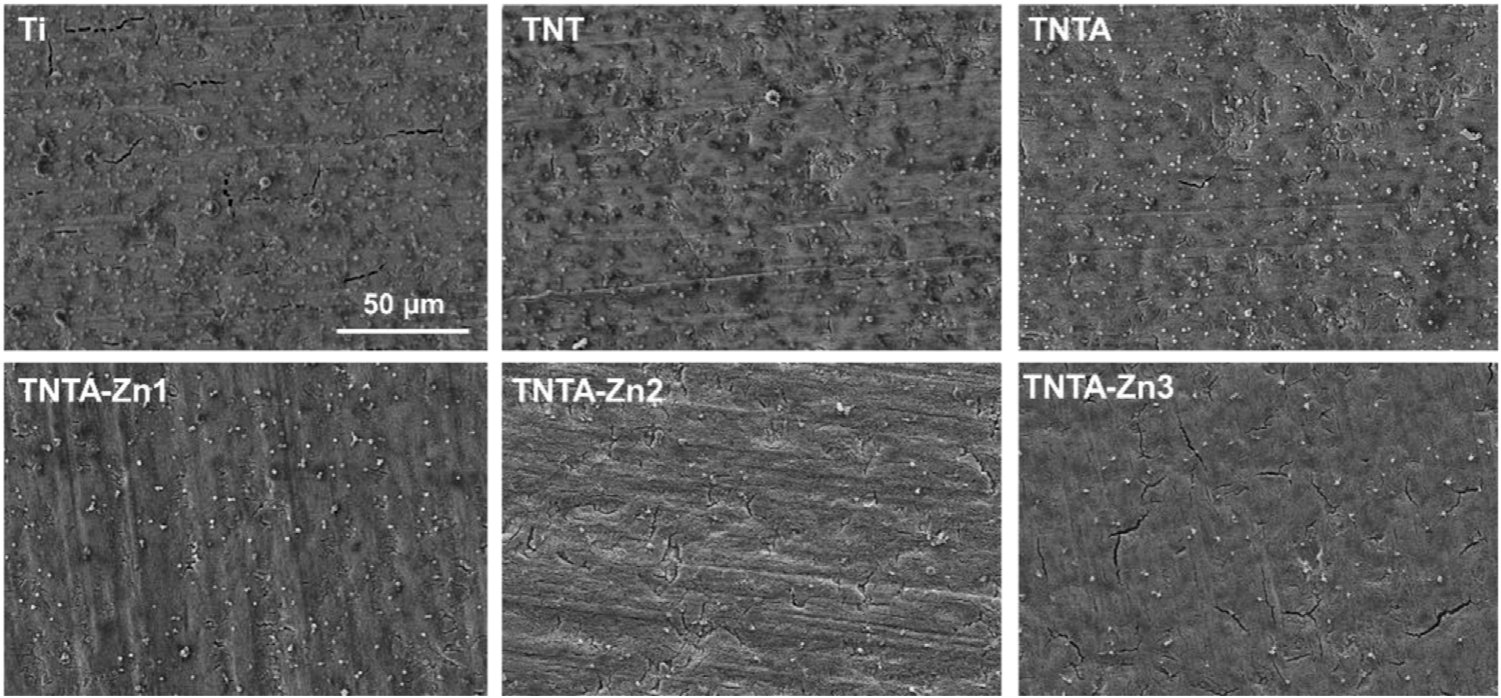
SEM images of the attached platelets on the different samples.

Platelets are one of the major components of the human blood. The aggregated and activated platelets can promote blood coagulation and even induce thrombus formation. Therefore, the materials with good blood compatibility should have the ability to maintain the normal physiological state of the platelets (Sheng et al., 2020). In this study, platelet adhesion and activation were first applied to investigate the blood compatibility of the modified titanium samples. As shown in Figure 5, it can be clearly seen that a lot of platelets were attached on the pristine titanium surface and some aggregated platelets were observed, indicating that the hemocompatibility of the pure titanium was limited and needed to be improved. After the anodization, the number of the attached platelets on the TNT surface decreased to some degree because the better hydrophilicity of the titanium oxide nanotubes can promote albumin adsorption and thus inhibit the platelet adhesion. After the annealing treatment, due to the improvement of the hydrophilicity, not only the number of the adhered platelets on the TNTA surface deceased significantly but also the aggregated platelets decreased obviously, suggesting that it has better ability to maintain the physiological state of the platelets than TNT. After the construction of the DA-Zn^2+^ coating on the TNTA surface, the number of the adhered platelets decreased significantly. Moreover, the number of the platelets on the surface decreased with the increase in the zinc concentration in the coatings. On the one hand, the selective albumin adsorption and the excellent hydrophilicity of the DA-Zn^2+^ coating can significantly inhibit the platelet adhesion. On the other hand, zinc ions released from the DA-Zn^2+^ coating can affect the activity of NOS enzyme and generate more NO signals, which can significantly prevent platelet adhesion and aggregation by inhibiting the thromboxane A2 (TXA2) receptor (Cortese et al., 2008).

**FIGURE 6:**
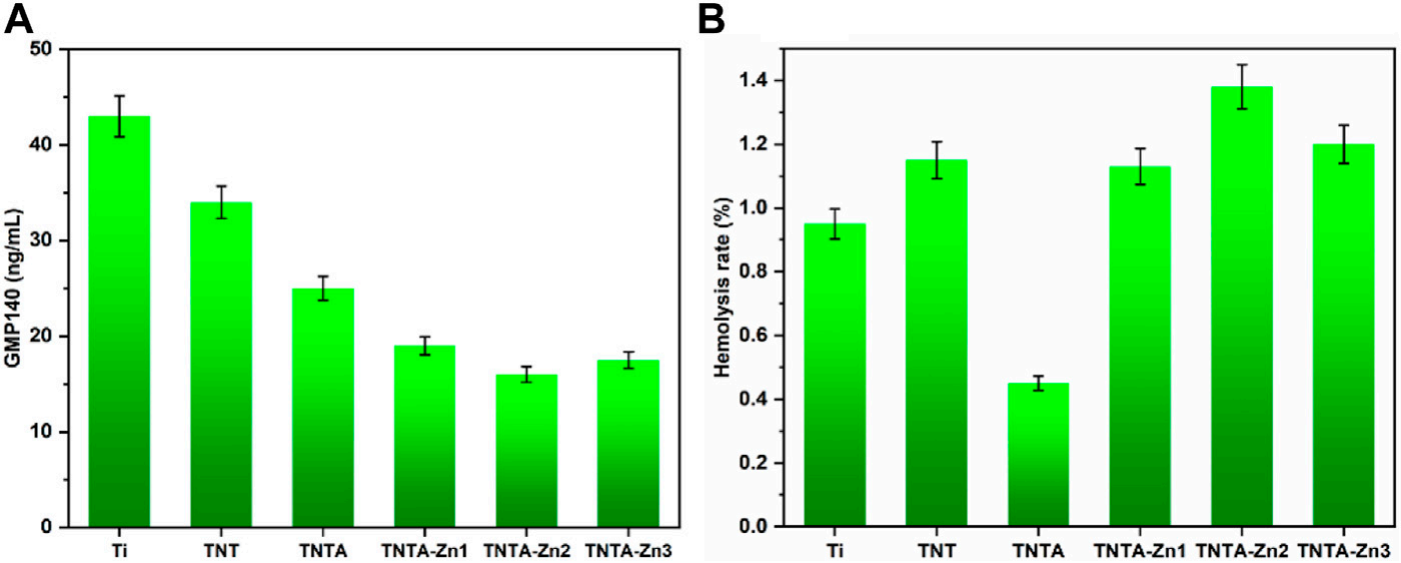
Platelet activation characterized by GMP140 **(A)** and the hemolysis rate of the different samples **(B)**.

The GMP140 expression of the attached platelets was further measured to characterize the degree of the platelet activation. It can be clearly seen from Figure 6A that the platelets attached on the pristine titanium surface were highly activated because of the largest GMP140 expression, suggesting that it was easy to induce human blood coagulation. The special nanotube structure can be produced by the anodic oxidation, and the resulting surface displayed better hydrophilicity; therefore, the platelet adhesion and activation were significantly reduced on the TNT surface. The annealed nanotubes can further inhibit the fibrinogen adsorption as compared to TNT, which could further contribute to prevent the platelet activation because the fibrinogen deposition on the surface of the platelet membrane binding receptor can lead to platelet activation and plasma coagulation. After the construction of the DA-Zn^2+^ coating, the platelet activation decreased further, which can be attributed to the fact that the activities of the platelets can be inhibited by zinc ions because the content of GMP140 decreased significantly; therefore, the DA-Zn^2+^-coated samples showed better anti-platelet activation. In addition, it can be seen that with the increase in the zinc concentration in the DA-Zn^2+^ coating, the values of GMP140 first decreased and then increased slightly, indicating that the large concentration of zinc ion may promote the platelet activation; however, there was no significant difference between TNT-Zn2 and TNTA-Zn3. Some studies have shown that the concentration of the external zinc ions in the range of 60–800 μM can promote platelet aggregation (Vu et al., 2013). In this study, the concentration of the released zinc ion (6.3–7.8 μM) from the samples at 14 days was far from the range of the platelet aggregation; thus, it could not cause platelet aggregation and activation.

The hemolysis rate represents one of the important factors to explore the interaction between the biomaterials and the erythrocytes. The hemolysis rate was further measured to characterize the influences of the DA-Zn^2+^ coating on the red blood cells. Normally, the hemolysis rate (HR) below 5% can be accepted for the application of the blood-contacting materials; otherwise, the material is not suitable for implants such as stent because serious hemolysis reaction can induce thrombosis by affecting the coagulation and other processes (Bartoli et al., 2018; Weisel and Litvinov, 2019). The hemolysis rates of the different samples are shown in Figure 6B. It can be clearly seen that the hemolysis rate of all samples was less than 5%, indicating that all samples can meet the requirement of relative standards and did not cause the hemolysis reaction. The surface of TNT had good hydrophilicity and a relatively larger hemolysis rate than the pure titanium, indicating that better hydrophilicity may not cause a smaller hemolysis rate, and other factors could also play an important role in affecting the hemolysis behaviors. It was considered that the surface morphology and the surface crystal structure had influences on the hemolysis behaviors, and the amorphous structure of TNT may not reduce the hemolysis reaction. However, excellent hydrophilicity can be achieved after the annealing treatment, and the hemolysis rate decreased significantly as compared to TNT, indicating that the improved hydrophilicity could reduce hemolysis. Therefore, we believed that hydrophilicity in a certain range may have better hemolytic properties. Moreover, the anatase crystal structure of TNTA also contributed to reduce the hemolysis reaction. After the fabrication of the dopamine–Zn^2+^ coating, the hydrophilicity increased further while the hemolysis rate also increased. It was considered that the coverage effect of the coating reduced the influence of the surface morphology and the crystal structure of the material on the hemolysis rate. Nevertheless, the hemolysis rate of all DA-Zn^2+^-modified nanotubes was very low and below 5%, which can meet the requirements of the blood-contacting materials.

### 3.4 Endothelial Cell Behaviors of the DA-Zn^2+^ Coating

**FIGURE 7:**
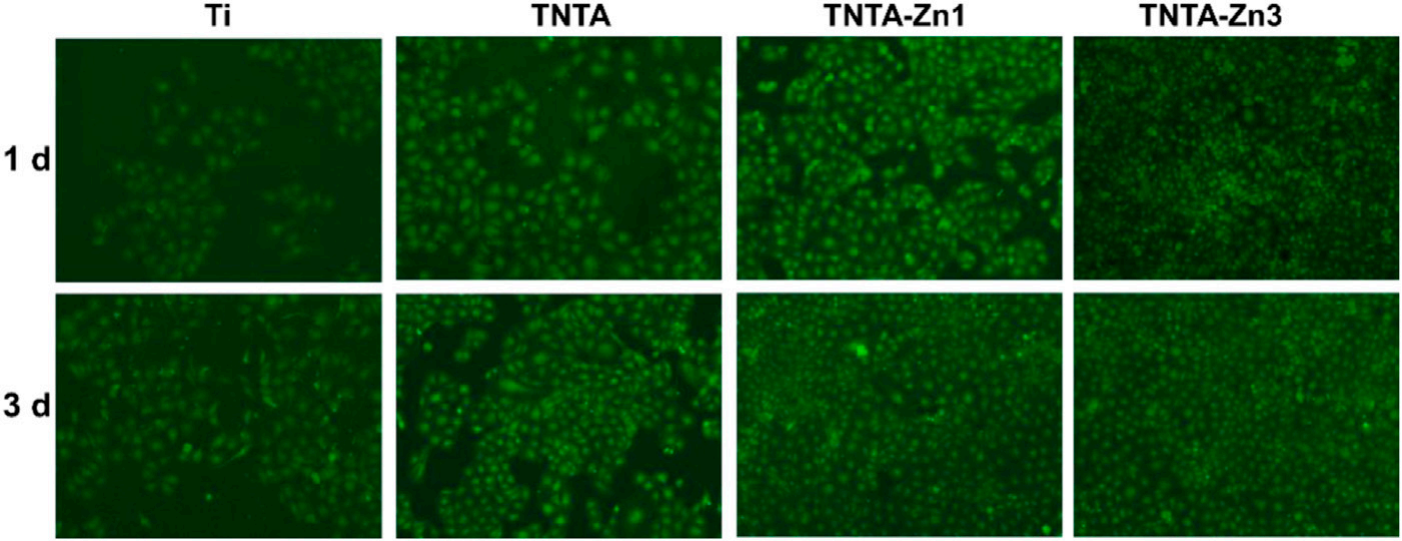
Fluorescence images of the endothelial cells adhered on the different samples.

Endothelial cell growth behaviors play an important role in controlling the angiogenesis of each organ system. Zinc is the second abundant trace element in the human body, and it is the basis of the normal cell structure and function (Kambe et al., 2015). In order to investigate the effects of the DA-Zn^2+^ coating on the endothelialization of the different samples, the cell adhesion behaviors on the surfaces were first studied by fluorescent staining, and the results are shown in Figure 7. It can be seen that whether they were cultured for 1 or 3 days, the cells on the pristine titanium surface were the least when compared to other samples. After the anodization and the following annealing treatment, the number of the endothelial cells on the TNTA surface increased significantly, indicating that the annealed nanotubes can effectively promote the endothelial cell adhesion and growth, which can be attributed to the excellent hydrophilicity and the specific surface nanotopography of TNTA. After the construction of the DA-Zn^2+^ coating, the number of the endothelial cells that adhered to the surface increased significantly, and the surface was almost completely covered by the endothelial cells, indicating that the DA-Zn^2+^ coating can further enhance the endothelial cell adhesion and growth. It has been reported that zinc is an essential trace element for cell growth. When the Zn^2+^ concentration is below 80 μM, it can promote the growth of the endothelial cells (Krones et al., 2005). In this study, the concentration of zinc ion (6.3–7.8 μM) released from the surface after 14 days was just within the range of promoting the growth of endothelial cells. Due to the large amount of zinc ions on the TNTA-Zn3 surface, endothelial cells can grow and adhere better on its surface.

**FIGURE 8:**
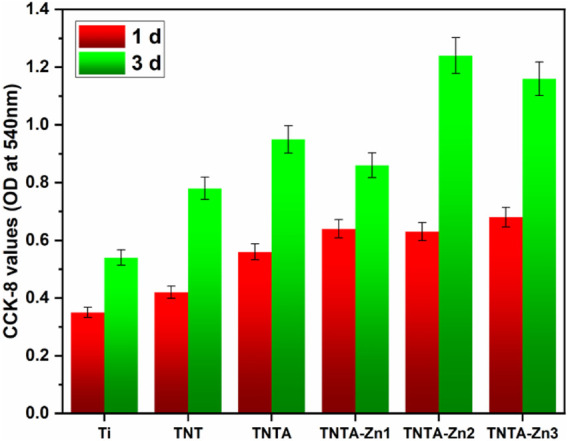
Proliferation behaviors of the endothelial cells on the different samples characterized by CCK-8.

The endothelial cell proliferation of the different samples was further characterized by CCK-8, and the results are shown in Figure 8. Some studies have shown that the titanium oxide nanotubes can enhance the endothelial cell growth because it is more conducive to protein, ion, and nutrient adsorption (Oh and Jin, 2006); therefore, the CCK-8 values of TNT and TNTA were increased when cultured for 1 and 3 days, respectively, indicating that both the anodized and the annealed nanotubes can promote cell proliferation. Zinc ions, being the second messenger of cells, can regulate the enzyme activity of the metabolism system in cells and control the proliferation, differentiation, and survival of cells (Levaot and Hershfinkel, 2018). According to the results shown in Figure 8, the CCK-8 values of the DA-Zn^2+^-coated surfaces were indeed higher than those of TNTA, indicating that the zinc ions released from the DA-Zn^2+^ coating can indeed promote the proliferation of the endothelial cells. Moreover, a small amount of fibrin adsorbed on the DA-Zn^2+^ coating could also be conducive to the expression of vascular endothelial growth factor (VEGF) of the endothelial cells (Nishikawa et al., 1998), leading to the improvement of cell proliferation. With the increase in zinc ions, more zinc ions can release from the DA-Zn^2+^ coating to further promote cell growth and generate more transporters and growth factors, and therefore enhance the proliferation of the endothelial cells. In addition, the hydroxyl and amine groups of the DA-Zn^2+^ coating on the surface can also recruit cell adhesion and enhance cell proliferation through non-receptor binding force with these functional groups. Consequently, the CCK-8 values of TNTA-Zn2 and TNTA-Zn3 were larger than those of TNT-Zn1 and TNTA.

### 3.5 Antibacterial Activity

**FIGURE 9:**
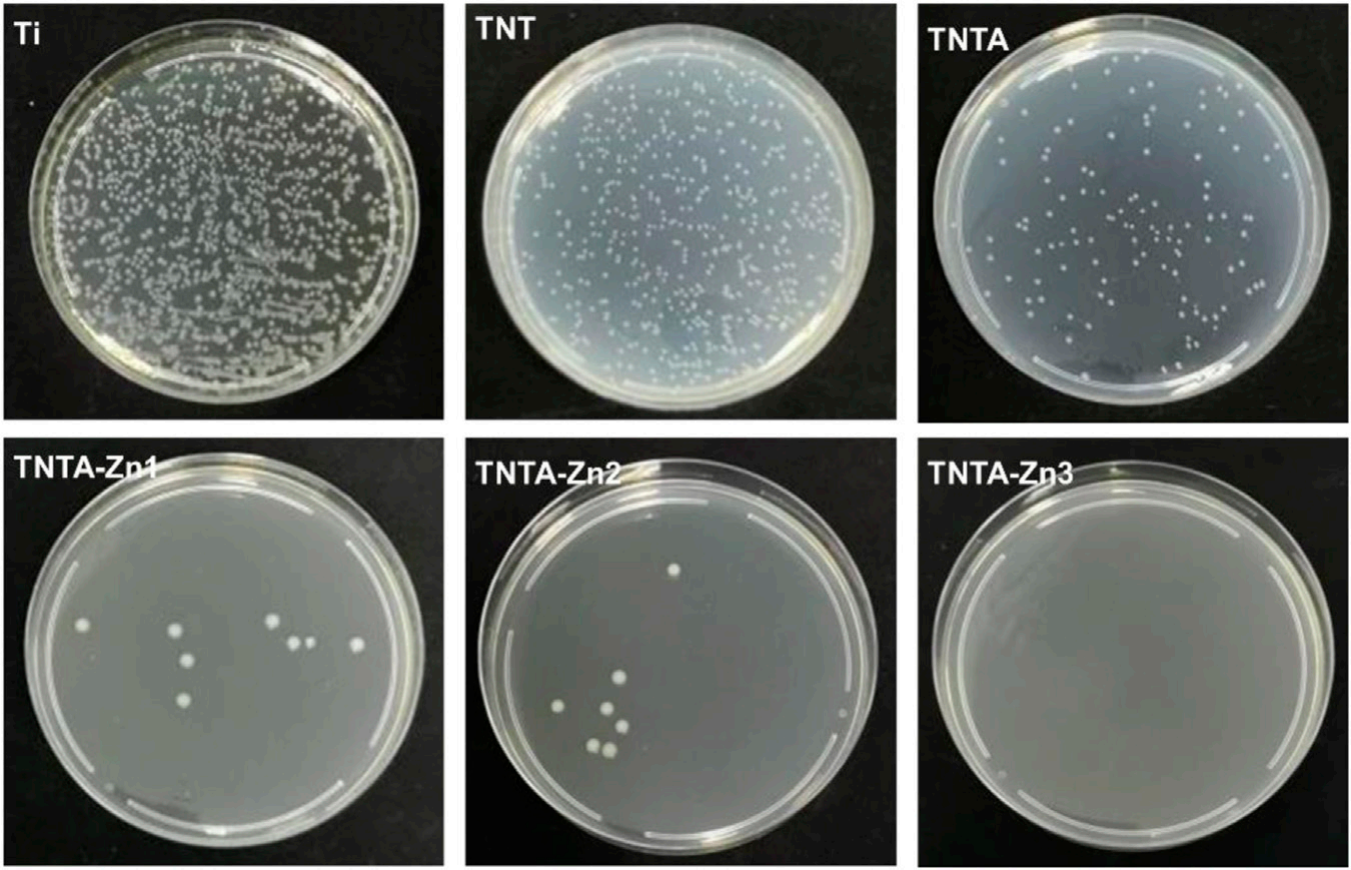
Antibacterial properties of the different samples using *Escherichia coli* as the model bacteria.

Biomaterial-centered infection is one of the main reasons for the failure of the implantable devices (Gristina, 1987). Zinc ions have been proved to have good antibacterial activities against Gram-positive (e.g., *Staphylococcus aureus* and *Staphylococcus epidermidis*) and Gram-negative bacteria (such as *Escherichia coli*) (Sirelkhatim et al., 2015). Accordingly, in this study, Gram-negative bacteria, *Escherichia coli*, was used as a test strain to evaluate the antibacterial properties of the DA-Zn^2+^-modified samples. As shown in Figure 9, it can be seen that the pure titanium, TNT, and TNTA had poor antibacterial activity, although the better hydrophilicity of TNT and TNTA can enhance the antibacterial activity to some degree. After the preparation of the DA-Zn^2+^ coating, the number of *Escherichia coli* colonies decreased significantly, indicating that the coating had excellent antibacterial properties. Zn^2+^ has excellent antibacterial activities, and it can inactivate the protein needed by bacteria and cause the condensation of DNA, finally resulting in the enhancement of the antibacterial activity of the biomaterials. Consequently, with the increase in the zinc ion concentration in the coating, the number of the observed colonies decreased significantly, and no colonies can be found on TNTA-Zn3.

## 4 Conclusion

Nanotube arrays with regular nanostructures were successfully constructed *in situ* on the titanium surface by anodic oxidation, which significantly improved the hydrophilicity of the titanium surface. The annealing treatment further improved the hydrophilicity of the nanotubes and changed the crystal structure of the nanotubes, leading to the selective adsorption of albumin, which contributed to improve the blood compatibility and cytocompatibility of titanium to endothelial cells to a certain extent. In order to further improve the biocompatibility and endow the material with good antibacterial properties, dopamine–Zn^2+^ coatings with the different zinc ion concentrations were successfully one-step-prepared on the surface of the annealed nanotube arrays. The zinc ions in the coating were released for more than 14 days, and the blood compatibility, endothelial cell adhesion and proliferation, and antibacterial properties of the material were significantly improved by the DA-Zn^2+^ coating. Therefore, the method used in the present study can be used to modify the titanium-based biomaterials to simultaneously improve the anticoagulants, cytocompatibility to endothelial cells, and antibacterial properties; it was envisioned that it can be applied in the fields of the blood-contacting materials or devices such as artificial heart valves and vascular stents.

## Data Availability

The raw data supporting the conclusion of this article will be made available by the authors, without undue reservation.
